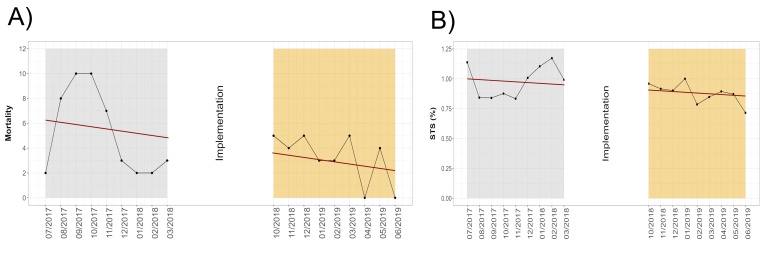# Data-driven coaching to improve statewide outcomes in CABG: before and after interventional study

**DOI:** 10.1097/JS9.0000000000001153

**Published:** 2024-02-13

**Authors:** Omar A.V. Mejia, Gabrielle B. Borgomoni, Fabiane Letícia de Freitas, Lucas S. Furlán, Bianca Maria M. Orlandi, Marcos G. Tiveron, Pedro Gabriel M de B e Silva, Marcelo A. Nakazone, Marco A. P de Oliveira, Valquíria P. Campagnucci, Sharon-Lise Normand, Roger D. Dias, Fábio B. Jatene

**Affiliations:** a Instituto do Coração (InCor), Hospital das Clínicas HCFMUSP, Faculty of Medicine, University of São Paulo; b Hospital Samaritano Paulista; c Hospital Paulistano; d Irmandade da Santa Casa de Misericórdia de Marília, Marília; e Hospital De Base de São José do Rio Preto, São José de Rio Preto; f Beneficência Portuguesa de São Paulo; g Irmandade da Santa Casa de Misericórdia de São Paulo, São Paulo, São Paulo, Brazil; h Department of Health Care Policy, Harvard Medical School; i Harvard Medical School, Boston, Massachusetts, USA

**Keywords:** coronary artery bypass grafting, database, operative mortality, postoperative complications, quality improvement initiatives

## Abstract

**Background:**

The impact of quality improvement initiatives program (QIP) on coronary artery bypass grafting surgery (CABG) remains scarce, despite improved outcomes in other surgical areas. This study aims to evaluate the impact of a package of QIP on mortality rates among patients undergoing CABG.

**Materials and methods:**

This prospective cohort study utilized data from the multicenter database *Registro Paulista de Cirurgia Cardiovascular II* (REPLICCAR II), spanning from July 2017 to June 2019. Data from 4018 isolated CABG adult patients were collected and analyzed in three phases: before-implementation, implementation, and after-implementation of the intervention (which comprised QIP training for the hospital team). Propensity Score Matching was used to balance the groups of 2170 patients each for a comparative analysis of the following outcomes: reoperation, deep sternal wound infection/mediastinitis ≤30 days, cerebrovascular accident, acute kidney injury, ventilation time >24 h, length of stay <6 days, length of stay >14 days, morbidity and mortality, and operative mortality. A multiple regression model was constructed to predict mortality outcomes.

**Results:**

Following implementation, there was a significant reduction of operative mortality (61.7%, *P*=0.046), as well as deep sternal wound infection/mediastinitis (*P*<0.001), sepsis (*P*=0.002), ventilation time in hours (*P*<0.001), prolonged ventilation time (*P*=0.009), postoperative peak blood glucose (*P*<0.001), total length of hospital stay (*P*<0.001). Additionally, there was a greater use of arterial grafts, including internal thoracic (*P*<0.001) and radial (*P*=0.038), along with a higher rate of skeletonized dissection of the internal thoracic artery.

**Conclusions:**

QIP was associated with a 61.7% reduction in operative mortality following CABG. Although not all complications exhibited a decline, the reduction in mortality suggests a possible decrease in failure to rescue during the after-implementation period.

## Graphical abstract

**Figure:**
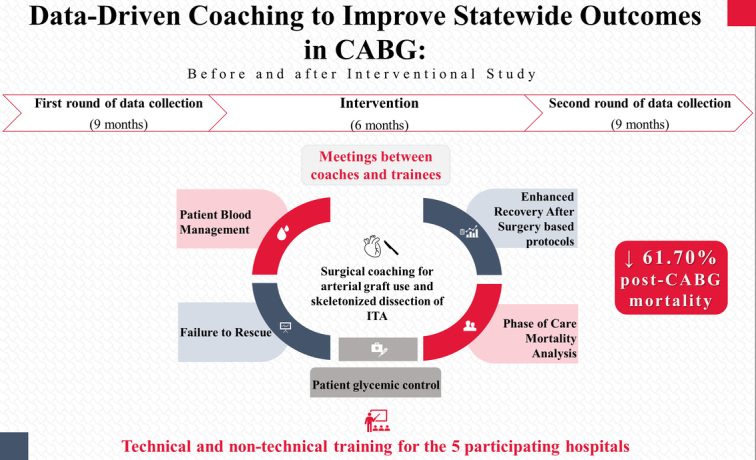

## Introduction

Highlights

- Quality improvement initiatives based on training in nonsurgical skills and surgical coaching can improve outcomes following coronary artery bypass grafting surgery.
- When comparing the pretraining and post-training periods in quality improvement initiatives, there was a 61.70% reduction in mortality rates following coronary artery bypass grafting surgery.
- The focus on training based on nonsurgical skills and surgical coaching may represent the frontier for achieving continuous improvement in the results of coronary artery bypass surgery.

In cardiac surgery (CS), the public disclosure of surgical outcomes has been a significant driver for quality improvement initiatives (QIP)^1^. This phenomenon led to the establishment of large databases, the presentation of results in an adjusted way, and the formulation of quality standards^2,3^. As a result, risk stratification and scores have become indispensable tools for monitoring outcomes.

Technical training and standardized perioperative care have contributed to continuous improvement, even in increasingly severe patients. Root cause analysis highlighted the need for nonsurgical skills training^4–6^ and surgical coaching^7,8^ for continuous progress. While surgical skills training was always prioritized, nonsurgical training became essential for better teamwork, task management, and effective response to complications. These ideas can be merged into quality programs focusing on data-driven improvements^4–6,8^.

For a long time, it was believed that surgical results depended solely and absolutely on the improvement of the surgical technique, where technical skills such as speed, assertiveness, and boldness would have a unique role in the results^9^. However, with the increase in the complexity of surgeries and the risk profile of patients, systemic interventions from high-reliability industries such as continuous team training^10^ and nontechnical skills training in surgical teams^11^ have become indispensable. Here, it is worth mentioning the use of surgical coaching as a learning strategy to improve human performance^12^.

Within a traditionally conservative setting like CS, the inclusion of training encompassing skills beyond surgical techniques poses significant challenges, essential for the successful implementation and sustainability of the new protocols^13,14^.

The aim of this study was to evaluate whether nontechnical data-driven interventions could be associated with a reduction of mortality and complications after CABG. Our hypothesis was that an educational program training care teams with issues that impact practice and combining coaching for the surgical team would help reduce mortality and complications after CABG.

## Methods

A prospective, observational, multicenter intervention study was conducted by the hospital coordinating the REPLICCAR Project. Between July 2017 and June 2019, five referral hospitals in São Paulo, Brazil, consecutively enrolled patients who underwent CABG (*N*=4018). The inclusion criteria were patients over 18 years old undergoing CABG, while exclusion criteria were an indication for combined surgery and emergency procedures.

All data obtained were entered into the REDCap platform, within an area created to the REPLICCAR II project, by health-trained professionals assigned to this task. All variables and outcomes followed the criteria and definitions from the STS Adult CS^15^ Database version 2.9^15^. Periodic quality audits were carried out to verify data accuracy, integrity, and consistency^16^.

As shown in Supplementary Figure 1 (Supplemental Digital Content 1, http://links.lww.com/JS9/B931), the study followed a specific timeline, beginning with a 9-month period of initial data collection (usual care). This was followed by a 6-month period that included data analysis to implement a package of improvements focused on training in nonsurgical skills and surgical coaching, followed by a new 9-month data collection period [before (*N*=1910) and after-implementation (*N*=1163) periods].

**Figure 1:**
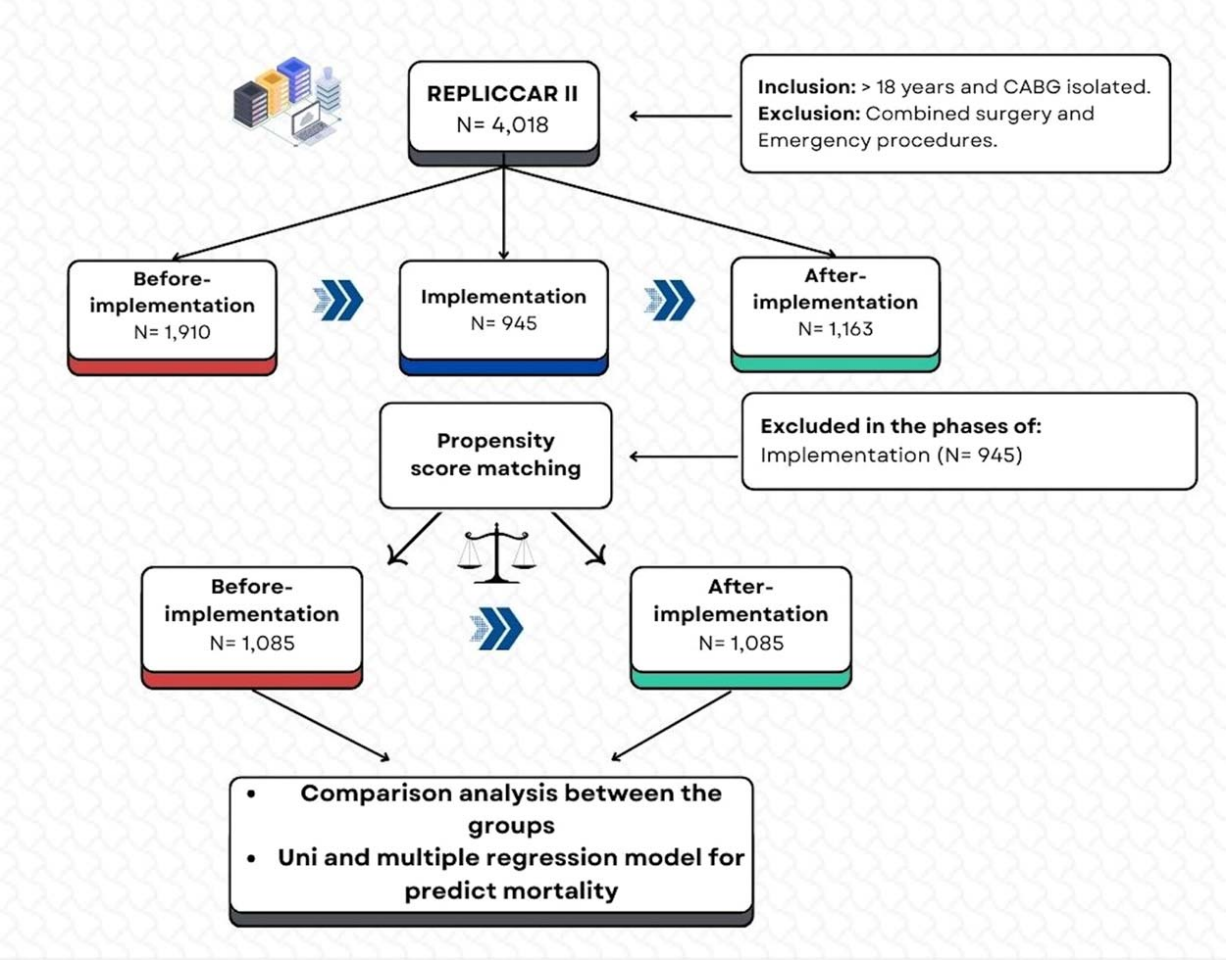
Study flowchart.

After carrying out the Propensity Score Matching (PSM), we compared the before and after-implementation periods (*N*=2170). It is important to mention that data from the implementation phase were not analyzed, as described in the study flowchart (Fig. 1). This study has been reported in line with the strengthening the reporting of cohort, cross-sectional, and case–control studies in surgery (STROCSS) criteria^17^ (Supplemental Digital Content 2, http://links.lww.com/JS9/B932).

### Interventions

**Figure 2:**
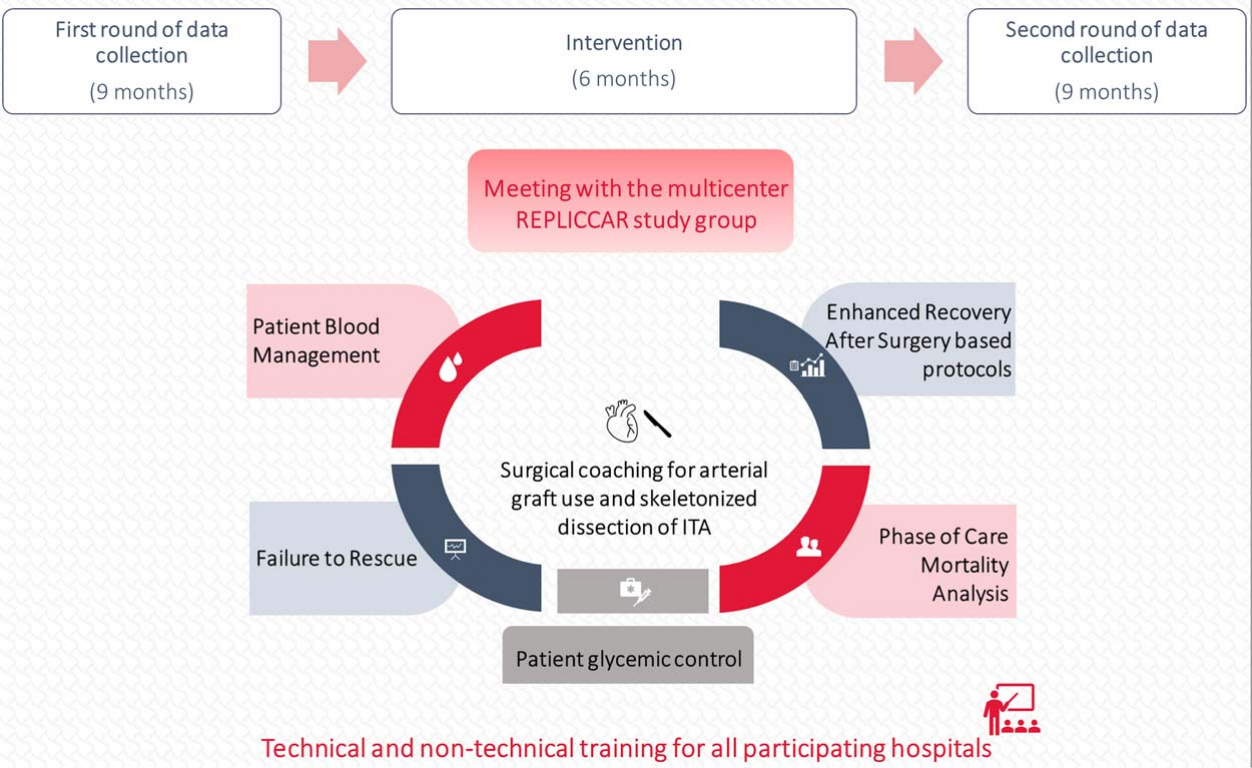
Methodology used for implementing a package of measures focusing on quality improvement initiatives.

After analyzing the data from the initial period, we implemented a package of measures focusing on QIP (Fig. 2). Thus, the chosen strategies encompassed the training of hospital teams in five nonsurgical skills: 1) Phase of Care Mortality Analysis (POCMA)^18^, 2) patient glycemic control^19^, 3) patient blood management^20^, 4) optimization of hospitalization times based on Enhanced Recovery After Surgery (ERAS) protocol^21,22^, and 5) impact of failure to rescue^23^. In addition, guidance based on surgical coaching was provided to enhance the use of arterial grafts and the rate of skeletonized dissection of the internal thoracic artery^24^.

An educational program was conducted at hospitals starting with visits to understand care workflows. Then, a six-part, 1 h online training on key topics was presented, half of which covered theoretical foundations and evidence, followed by discussions on improving hospital outcomes using specific data, with multiprofessional leaders of each hospital.

After the implementation of this package of measures, a training program was structured for the CS care line at each participating hospital, involving a 10-day immersion at each facility, including on-site visits and both synchronous and asynchronous classes.

The study executive committee, together in collaboration with the principal investigators from each hospital, evaluated and certified the readiness of each facility for the after-implementation phase. Regarding surgical coaching, each surgical team was able to increase the use of arterial grafts, including the double internal thoracic artery and radial artery grafts^24,25^, and increase the rate of skeletonized dissection of the internal thoracic artery. The orientations provided on surgical coaching were structured based on the concepts of the Michigan model^25^. Throughout the after-implementation period, periodic meetings were held with the research committee of each center to monitor the teams’ performance. It is important to note that it was an indispensable requirement to enter the study that each participating hospital was committed to implement the strategies outlined by the executive committee of the REPLICCAR II project. Thus, all five participating centers signed a consent agreement with the research committee of the coordinating center.

It is worth emphasizing that the leadership of each hospital’s board of directors endorsed the design of the REPLICCAR II project. The primary aim of the project was to enhance the outcomes of myocardial revascularization surgeries through a comprehensive training program encompassing both technical and nontechnical aspects of healthcare delivery. This training focused on optimizing perioperative care without requiring substantial investments in additional staff or physical hospital infrastructure.

The training process involved an initial general meeting with the participating centers to present the data and its association with the outcomes. Subsequently, six training sessions were conducted for each theme, tailored to the unique characteristics of each hospital. After the completion of training for each center and validation by their respective principal researchers, the project’s executive committee approved the training program and initiated the second phase of data collection.

### Outcomes

Nine outcome variables were used to compare the preimplementation and after-implementation phases: reoperation for bleeding, deep sternal wound infection ≤30 days, cerebrovascular accident, acute kidney failure, ventilation time >24 h, length of stay <6 days, length of stay >14 days, morbidity and mortality, and operative mortality.

The outcomes were defined as follows: 1) Morbidity: development of a composite outcome (regardless of the number of associations), including cerebrovascular accident, acute kidney failure, prolonged ventilation, deep sternal wound infection, and reoperation for bleeding; and 2) Operative mortality: defined as death occurring during the hospitalization in which the operation was performed or all deaths, regardless of cause, that occurred after hospital discharge but before the end of the 30th postoperative day.

### Statistical methods

The statistical analyses were conducted using R software version 4.0.2.

Descriptive statistics for continuous variables were expressed in terms of summary measures (mean, median, SD, and quartiles), while categorical variables were expressed as percentages. Due to missing data, the percentages were calculated based on the number of responses obtained rather than the total number of patients.

PSM was used to pair the groups using the GenMatch function (Supplementary Table 1, Supplemental Digital Content 3, http://links.lww.com/JS9/B933), available in the MatchIt package of R software, and its quality was verified by using the standardized mean difference (SMD) method. The variables used for matching included age, sex, hospital admission status, diabetes control, and the Society of Thoracic Surgeons (STS) risk score for mortality.

Continuous variables from the two groups were compared using the *t*-test for normally distributed variables (Anderson-Darling test), and nonparametric tests were applied for the other variables. Mann–Whitney test was used for homogeneous variables, and the Brunner–Munzel test was used for heterogeneous variables. Categorical variables were analyzed using Fisher’s exact test or the *χ* ^2^ test. Two-tailed hypotheses were considered. Furthermore, the constructed confidence intervals have a 95% confidence level.

The primary analysis aimed to assess the effect of the intervention on discrepancy rates, with ancillary analyses conducted to determine mechanisms of action.

For the associations between explanatory variables and outcomes, the logistic regression model was used. Significant variables identified in the simple model were then used in the forward stepwise regression to build the multiple model. The multiple model was evaluated using the Hosmer–Lemeshow test, and C statistics were calculated to assess model performance.

## Results

After PSM, 1085 patients each were evaluated both before and after the implementation of the package of measures (*N*=2170, Table 1). In the before-implementation phase, patients had a higher proportion of females (*P*=0.013), elevated glycosylated hemoglobin levels (*P*=0.003), increased blood glucose levels (*P*=0.019), and a higher prevalence of Canadian Cardiovascular Society (CCS) angina class III (*P*=0.010). After-implementation patients had a higher BMI (*P*=0.020), family history of coronary artery disease (*P*<0.001), and a higher incidence of CCS class IV (*P*=0.010).

**Table 1:** Preoperative characterization of REPLICCAR II patients after propensity score matching.

	Before-implementation (*N*=1085)	After-implementation (*N*=1085)	
Variable	*N* (%)	*N* (%)	*P*
Sex			0.013
Female	293 (27%)	242 (22.3%)	
Male	792 (73%)	843 (77.7%)	
Age, mean±SD	63.57±9.19	63.08±9.07	0.231
BMI, mean±SD	27.12±4.15	27.55±4.43	0.020
Glycosylated hemoglobin, mean±SD	6.93±1.67	6.66±1.47	0.003
Creatinine, mean±SD	1.24±1.09	1.25±1.06	0.896
Blood glucose (mg/dl), mean±SD	145.71±65.77	136.40±58.18	0.019
Hemoglobin (mg/dl), mean±SD	13.49±1.77	13.49±1.74	0.94
Left ventricular ejection fraction (%), mean±SD	56.34±12.92	51.90±19.75	0.007
Acute myocardial infarction	568 (52.35%)	576 (53.09%)	0.763
Cerebrovascular accident	40 (3.69%)	55 (5.07%)	0.141
Systemic arterial hypertension	957 (88.2%)	972 (89.59%)	0.339
Diabetes mellitus	543 (50.05%)	558 (51.43%)	0.548
CCS angina classification (2 weeks before the procedure)			0.010
I	525 (48.39%)	495 (45.62%)	
II	253 (23.32%)	313 (28.85%)	
III	206 (18.99%)	168 (15.48%)	
IV	101 (9.31%)	109 (10.05%)	
NYHA classification			0.135
I	720 (66.36%)	752 (69.31%)	
II	183 (16.87%)	190 (17.51%)	
III	143 (13.18%)	111 (10.23%)	
IV	39 (3.59%)	32 (2.95%)	
STS mortality risk (%), mean±SD	0.91±0.86	0.89±0.80	0.508
Number of vessels affected			0.402
One	38 (4.5%)	34 (3.32%)	
Two	129 (15.28%)	169 (16.49%)	
Three	677 (80.21%)	821 (80.1%)	
Type of surgery			1.000
Elective	610 (56.22%)	610 (56.22%)	
Urgent	254 (23.41%)	254 (23.41%)	
Transfer from another hospital	221 (20.37%)	221 (20.37%)	

CCS classification: Canadian Cardiovascular Society classification for angina; NYHA classification: New York Heart Association functional classification; REPLICCAR II: Registro Paulista de Cirurgia Cardiovascular II; STS: Society of Thoracic Surgeons.

Regarding the intraoperative period (Table 2), it was observed that patients followed up in the after-implementation period had a prolonged cardiopulmonary bypass time (*P*<0.001), cross-clamp time (*P*<0.001), and surgical time (*P*<0.001) compared to the before-implementation group. Additionally, there was a greater use of arterial grafts [internal thoracic (*P*<0.001) and radial (*P*=0.038)], a higher rate of skeletonized dissection of the internal thoracic artery, and a greater frequency of extubation in the operating room (*P*<0.001).

**Table 2:** Intraoperative variables of REPLICCAR II patients after propensity score matching.

	Before-implementation (*N*=1085)	After-implementation (*N*=1085)	
Variable	*N* (%)	*N* (%)	*P*
Use of cardiopulmonary bypass	977 (90.05%)	991 (91.34%)	0.337
Cardiopulmonary bypass time (min), mean±SD	74.80±29.64	80.98±27.96	<0.001
Cross-clamp time (min), mean±SD	56.11±24.33	62.19±23.39	<0.001
Higher blood glucose, mean±SD	179.85±56.80	195.94±62.64	<0.001
Lower hemoglobin, mean±SD	9.26±2.21	9.18±2.40	0.101
Packed red blood cells transfusion	187 (17.24%)	179 (16.5%)	0.688
Use of left internal thoracic artery	1042 (96.04%)	1039 (95.76%)	0.745
Type of dissection and preparation of left internal thoracic artery			< 0.001
Pedicled	718 (66.18%)	623 (57.42%)	
Skeletonized	324 (29.86%)	416 (38.34%)	
Use of right internal thoracic artery	98 (9.03%)	172 (15.85%)	< 0.001
Type of dissection and preparation of right internal thoracic artery			<0.001
Pedicled	38 (38.76%)	39 (22.68%)	
Skeletonized	60 (61.24%)	133 (77.32%)	
Use of radial artery	30 (2.76%)	49 (4.52%)	0.038
Surgery duration (hours), mean±SD	4.21±1.39	4.96±1.49	<0.001
Extubation in the operating room	21 (1.94%)	110 (10.14%)	<0.001

REPLICCAR II: Registro Paulista de Cirurgia Cardiovascular II.

Table 3 compares postoperative variables, addressing outcomes and the length of hospital stay. After the implementation of the data driven interventions were observed a significant reduction of operative mortality (*P*=0.046, Supplementary Figure 2, Supplemental Digital Content 4, http://links.lww.com/JS9/B934, where the preimplementation and after-implementation graphs represent the reduction in observed mortality [a] in patients with the same mortality risk expected by STS [b]), the first outcome of this present analysis. On the same hand, the postimplementation period had a decrease of deep sternal wound infection/mediastinitis (*P*<0.001), sepsis (*P*=0.002), ventilation time (*P*<0.001), prolonged ventilation (*P*=0.009), postoperative peak blood glucose (*P*<0.001), and prolonged and total hospitalization time (*P*=0.003 and *P*<0.001, respectively).

**Table 3:** Outcome variables and postoperative evolution of REPLICCAR II patients after PSM.

	Before-implementation (*N*=1085)	After-implementation (*N*=1085)	
Variable	*N* (%)	*N* (%)	*P*
Need for an intra-aortic balloon pump			0.802
Preoperative intra-aortic balloon pump	37 (62.71%)	29 (67.44)	
Intraoperative intra-aortic balloon pump	9 (15.25%)	7 (16.28%)	
Preoperative intra-aortic balloon pump	13 (22.03%)	7 (16.28%)	
Cerebrovascular accident	21 (1.94%)	12 (1.11%)	0.174
Atrial fibrillation	143 (13.18%)	184 (16.96%)	0.016
Deep sternal wound infection/mediastinitis	48 (4.42%)	18 (1.66%)	<0.001
≤30 postoperative days	46 (4.24%)	15 (1.38%)	
>30 postoperative days, during hospitalization	2 (0.18%)	3 (0.28%)	
Sepsis	60 (5.53%)	30 (2.76%)	0.002
Acute kidney injury	66 (6.08%)	97 (8.94%)	0.014
Reoperation for bleeding with or without cardiac tamponade	11 (1.01%)	11 (1.01%)	1.0
Multiorgan dysfunction	11 (1.01%)	7 (0.65%)	0.479
Pleural effusion with indication for drainage	22 (2.03%)	15 (1.38%)	0.32
Pneumonia	41 (3.78%)	45 (4.15%)	0.442
Pneumothorax with indication for intervention	12 (1.11%)	10 (0.92%)	0.831
Packed red blood cells transfusion	163 (15.21%)	219 (20.6%)	0.001
Ventilation time (hours), median (interquartile range)^a^	9.26±4.89	7.98±4.98	<0.001
Ventilation time >24 h	61 (5.62%)	35 (3.23%)	0.009
Reintubation	35 (3.23%)	34 (3.13%)	1
Creatinine (mg/dl), mean±SD	1.52±1.38	1.55±1.43	0.149
Hemoglobin before hospital discharge, mean±SD	10.12±1.37	10.61±3.31	0.075
postoperative peak blood glucose, mean±SD	188.44±58.29	172.57±51.34	<0.001
Left ventricular ejection fraction (%), mean±SD	52.96±13.77	46.22±23.34	0.085
ICU readmission	49 (4.52%)	35 (3.23%)	0.148
Length of ICU stay (hours)^a^	75.67±41.91	77.31±39.10	0.003
Postoperative length of stay (days), mean±SD	8.04, ±3.72	7.83±3.58	0.051
Total hospitalization time (days), mean±SD	12.73±5.81	11.66±5.68	<0.001
Length of hospital stay <6 days	43 (3.96%)	43 (3.96%)	1.000
Length of hospital stay> 14 days	304 (28.02%)	243 (22.4%)	0.003
Hospital readmission up to 30 days after surgery	42 (3.87%)	34 (3.13%)	0.414
Morbidity	155 (14.29%)	137 (12.63%)	0.285
Morbidity and mortality	170 (15.67%)	141 (13%)	0.086
Operative mortality	47 (4.33%)	29 (2.67%)	0.046

a Associated with additional time related to readmission to the unit.

REPLICCAR II, *Registro Paulista de Cirurgia Cardiovascular II*.

To identify factors associated with the outcome of operative mortality, univariate regression analysis was used to identify variables correlated with the event (Supplementary Table 2, Supplemental Digital Content 5, http://links.lww.com/JS9/B935). Subsequently, from the data obtained in the analysis, multivariate regression was used to create a multiple model (Table 4).

**Table 4:** Multiple model.

Explanatory variable	OR	Inf 95% CI (OR)	Sup 95% CI (OR)	*P*
After-implementation of the package of measures	0.549	0.304	0.990	0.046
Age	1.062	1.027	1.098	<0.001
CPB time (min)	1.012	1.002	1.022	0.007
Need for an intra-aortic balloon pump	7.008	3.515	13.970	<0.001
Total postoperative pulmonary ventilation time	1.081	1.036	1.129	<0.001
ICU readmission	2.386	1.053	5.421	0.037
Kidney failure	12.846	7.149	23.082	<0.001

CPB, cardiopulmonary bypass.

The multiple model analysis yielded several important findings:

1. The study period was statistically relevant for patients, where the likelihood of death after CABG was 1.821 (1/0.549) times higher in the before-implementation period of the package of measures compared to the after-implementation period (*P*=0.046).
2. For every increase in the patient’s age, the likelihood of death increased by 1.062 times (*P*<0.001).
3. For each minute of CPB, the likelihood of death increased by 1.012 times (*P*=0.007).
4. The need for an intra-aortic balloon pump increased the likelihood of death by 7.008 times compared to those who did not require it (*P*<0.001).
5. Patients with kidney injury in the postoperative period were 12.846 times more likely to die (*P*<0.001).
6. Patients who required readmission to the ICU were 2.389 times more likely to die compared to those who did not require readmission (*P*=0.037).
7. With each additional hour of postoperative pulmonary ventilation, the likelihood of death increased by 1.081 times (*P*<0.001).

**Figure 3:**
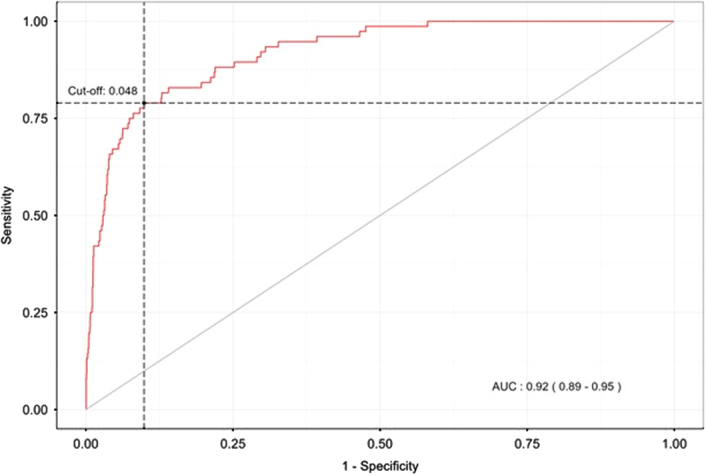
ROC curve of the model.

This multiple model for predicting mortality risk after CABG surgery was validated through calibration using the Hosmer–Lemeshow test (*P*=0.743) and discrimination with an area under the receiver operating characteristic (ROC) curve of 0.92 (Fig. 3).

## Discussion

Our study brings an unprecedented contribution, as it is the first to demonstrate a 61.7% reduction in mortality after CABG through the implementation of training in nonsurgical skills and the utilization of surgical coaching. Although existing evidence suggests that these measures can enhance surgical team performance and improve outcomes in other specialties^7,8^, the scientific literature still lacks evidence in cardiovascular surgery.

In this aspect, the establishment of a registry is pivotal in implementing QIP^26^. The release of surgical results in New York State has historically driven the emergence of large records in CS^1^, reducing hospital mortality by 65.7%. Over time, hospitals began to build databases and implement quality programs, resulting in significant improvements in outcomes^27–33^.

Our research group collaborated in establishing the pioneering REPLICCAR database^34,35^ in Brazil. This initiative has brought the first reports on the implementation of quality initiatives in our setting^36–39^. Participating centers in REPLICCAR embraced quality programs, including the largest public hospital for cardiac surgeries in a developing country, which achieved a significant reduction in mortality, and a private hospital that achieved a significant reduction in hospitalization times^28,40^.

The second REPLICCAR project had two objectives: upgrading our data collection for a quality registry^16^ and creating training strategies for better surgical outcomes based on the data collected during the before-implementation phase. Based on the data, the committee decided to provide training to hospital teams in nontechnical subjects: root cause analysis of mortality with POCMA^41^, glycemic control^19^, blood management^36,37,42^, ERAS protocol timing optimization^22,43,44^, reduction of failure to rescue^45,46^, and orientation based on surgical coaching for artery dissection and use of arterial grafts^47–49^.

The incorporation of training in nonsurgical skills and surgical coaching, although complementary to technical training in reducing adverse events^50^, is still rare and dispersed in hospitals^4,6–8,51^. However, this methodology has been adopted in centers in the United Kingdom, Australia, Canada, Japan, and Denmark^52,53^.

Root cause analysis of patients who have progressed to death helps to identify opportunities for improvement in perioperative care^40^. One of the most used instruments is the POCMA^41^, which helped us to understand areas where each center could improve and add efforts to reduce complications and, especially, deaths, as shown in other settings^18^.

Regarding glycemic control, patients in the after-implementation phase exhibited improved preoperative glycemic (*P*=0.009) and glycated hemoglobin (*P*=0.003) levels compared to patients in the before-implementation phase. Even though this was temporarily reversed during the intraoperative period (*P*<0.001), postoperative glycemic values returned to being significantly better in the after-implementation phase (*P*<0.001), which describes the teams’ proficiency in this linear control.

Our analysis showed that preimplementation and after-implementation patients had similar presurgery hemoglobin levels. However, after-implementation patients had slightly lower intraoperative hemoglobin, possibly leading to more red blood cell (RBC) transfusions postsurgery (*P*<0.001). Conversely, hemoglobin values were slightly higher before hospital discharge (*P*=0.075), which may have raised the postoperative kidney injury rates in after-implementation patients (*P*=0.014), as previously reported in the literature^54,55^. Notably, after the latest evidence regarding the criteria for RBC transfusion, we shifted from a restrictive policy^42^ to a rational transfusion model^56^. Despite a 20.6% transfusion rate in the after-implementation period, it is below the average of American centers^57^.

Based on ERAS protocol, our goal was to achieve immediate extubation and shorter hospital stays. In the after-implementation phase, the rate of extubation in the operating room increased (*P*<0.001), reducing extubation times and prolonged ventilation. Although there were no differences in ICU times and postoperative times in the after-implementation phase, total hospitalization times decreased (*P*<0.001).

This association was discussed in our recent study^58^. After-implementation phase saw longer cross-clamp and CPB times, in addition to prolonged operating room stay, likely due to the increased arterial graft use.

The failure to rescue concept introduced a new and well-defined quality metric for the teams: a parameter that evaluates the rapid response of teams in preventing death among patients who develop complications after surgery^45,46^. In our study, we can state that, in the after-implementation phase, there was no overall decrease in all complications (*P*=0.285) and even some complications such as atrial fibrillation (*P*=0.016) and acute kidney injury (*P*=0.014) increased during this phase. As hospital mortality decreased in the after-implementation phase, we can hypothesize that there was a decrease in failure to rescue during this period.

Regarding orientations based on surgical coaching, the emphasis was on two actions: increasing the use of arterial grafts and promoting the rate of skeletonized dissection of the internal thoracic artery. Comparing the preimplementation and after-implementation periods, we identified a significant increase in the use of arterial grafts (right internal thoracic artery, *P*<0.001 and radial graft, *P*=0.038), as well as in the rate of skeletonized dissection of the internal thoracic artery (*P*<0.001).

The increase in the rate of skeletonized dissection of the internal thoracic artery may have contributed to the decrease in the rate of deep sternal wound infection (*P*<0.001) observed in the after-implementation phase, consistent with several publications. On the other hand, the increased use of arterial grafts aligns with current best practices, as these grafts offer advantages such as longer patient survival in the long term, a lower incidence of complications, and better graft patency. These benefits have encouraged teams to use them, despite the greater technical difficulty and the consequent prolongation of surgical times^47,49,59^.

To validate the impact of the implementation of these QIP measures, we built a multiple model to identify variables that predict mortality in the registry. The before-implementation period was a predictor of mortality, increasing the likelihood of death by 1821 times. Other well-known predictive variables included increased age (OR: 1.062, CI: 1.027–1.098)^60,61^, longer CPB time (OR: 1.012; CI: 1.002–1.02; *P*=0.007)^62^, need for an intra-aortic balloon pump (OR: 7.008, CI: 3.515–13.970; *P*<0.001)^63,64^, ICU readmission (OR: 2.389, CI: 1.053–5.421)^65,66^, total pulmonary ventilation time (OR: 1.081, CI: 1.036–1.129)^67^, and postoperative kidney injury rate (OR: 12.846, CI: 7.149–23.082)^68–70^.

The internal validation of the multiple model demonstrated its accuracy in predicting mortality risk after CABG surgery. However, it is important to note that external validations are essential to confirm its efficiency and enable large-scale use. A follow-up analysis of those groups is being conducted by the authors and will be analyzed in the next year for publication. For instance, the successful results presented were due to having expanded training for the entire surgical and multidisciplinary team involved in patient care for CABG, which goes beyond the surgical technique, and impacts the system.

### Study limitations

Our study has some limitations.

1. Despite being a nonrandomized study, the data were analyzed from a prospective, multicenter registry (funded by the government and audited by an international institution), with a before and after-implementation analysis and risk adjustment of populations using PSM. Data accuracy was ensured through a structured database, validated variables, and quality audits^11^.
2. The before-after design does not consider secular trends. However, no other significant changes in the clinical practice of these hospitals were recorded or identified.
3. Training based on nonsurgical skills and surgical coaching was conducted by experts through interviews and classes. Although there was no measurement of the impact of these interventions, the participating institutions committed to following guided practices. The implementation of REPLICCAR brought opportunities for improvement in our CS scenario. It has enabled continuous monitoring of our practice over time, as well as process control and monitoring of risk factors. We understand that the enhancement of quality in CS implies perioperative care optimization. Multiple regression analysis confirmed that the before-implementation period, age, CPB time, need for an intra-aortic balloon pump, ICU readmission, kidney injury, and pulmonary ventilation time were predictors of operative mortality with an area under the ROC curve of 0.96%.

## Conclusions

The implementation of this statewide QIP, based on strategies from an analysis of the initial registry collection, was associated with a 61.70% reduction in mortality after CABG. It is noteworthy that this reduction in operative mortality, and not necessarily in other complications after CABG, may be attributed to a decrease in failure to rescue; however, further analyses should clarify this hypothesis.

## Ethical approval

The REPLICCAR II project was approved by the Research Ethics Committee (CAPPesq) of the Hospital das Clínicas of the University of São Paulo, under number 4506/17/006. The REPLICCAR II registry was approved for analysis by Harvard Medical School (DUA19-1460). Informed consent was waived in the initial data collection due to the research design methodology applied to the project. The project is registered at ClinicalTrials.gov as ‘The REPLICCAR Registry and The Statewide Quality Improvement Initiative’, ID: NCT05363696.

## Sources of funding

Approved under process No. 16/15163-0 for funding by the São Paulo Research Foundation (FAPESP), in partnership with the São Paulo State Department of Health (SES-SP), the Brazilian Ministry of Health (MS) and the Brazilian National Council for Scientific and Technological Development (CNPq), in compliance with the FAPESP/2016 Notice – Research for SUS: Shared Management in Health PPSUS – SP FAPESP/SES-SP/MS/CNPq (FAPESP Call 17/2016).

## Author contribution

O.A.V.M.: conceptualization and program administration, data collect, data analysis, roles/writing – original draft, review and editing; G.B.B.: data collect, data curation, data analysis, roles/writing – original draft, review and editing; F.L.d.F.: data analysis, roles/writing – original draft, review and editing; L.S.F.: data collect; B.M.M.O.: data curation; M.G.T.: data collect; P.G.M.d.B.e.S.: data collect; M.A.N.: data collect; M.A.P.de.O.: data collect; V.P.C.: data collect; S.-L.N.: data curation; R.D.D.: review and editing; F.B.J.: conceptualization and program administration, review and editing. All authors approved the final version of this manuscript and agreed to the submission.

## Conflicts of interest disclosure

The authors declare that they have no financial conflict of interest with regard to the content of this report.

## Research registration unique identifying number (UIN)

1. Name of the registry: The REPLICCAR Registry and The Statewide Quality Improvement Initiative.
2. Unique identifying number or registration ID: NCT05363696.
3. Hyperlink to your specific registration (must be publicly accessible and will be checked): https://clinicaltrials.gov/ct2/show/nct05363696.

## Guarantor

Omar Asdrúbal Vilca Mejia, 44 Dr. Enéas de Carvalho Aguiar Avenue, Block II – 2nd floor, 11 room – Postal code: 05403-000, São Paulo, SP, Brazil. E-mail: omar.mejia@incor.usp.br.

## Data availability statement

The data underlying this study cannot be made available due to ethical restrictions; patients did not consent to their de-identified data being publicly shared. De-identified data can be made available to qualified researchers under their responsibility and assuming the penalties if public disclosure of the data. Data requests should be sent to Renata do Val, Director of the Scientific Committee, Ethics Committee of the Heart Institute—University of São Paulo (renata.doval@incor.usp.br, http://www.incor.usp.br/sites/incor2013/index.php/equipe/16-pesquisa/comissao-cientifica/158-fale-conosco [1]) or Prof. Dr. Alfredo José Mansur, Coordinator, Comissão de Ética para Análise de Projetos de Pesquisa—CAPPesq (cappesq.adm@hc.fm.usp.br, http://www.hc.fm.usp.br/index.php?option=com_content&view=article&id=243:comissao-de-etica-para-analise-de-projetos-de-pesquisa-do-hcfmusp&catid=23&Itemid=229 [2]).

## Provenance and peer review

Not commissioned, externally peer-reviewed.

## Supplementary Material

**Figure:**
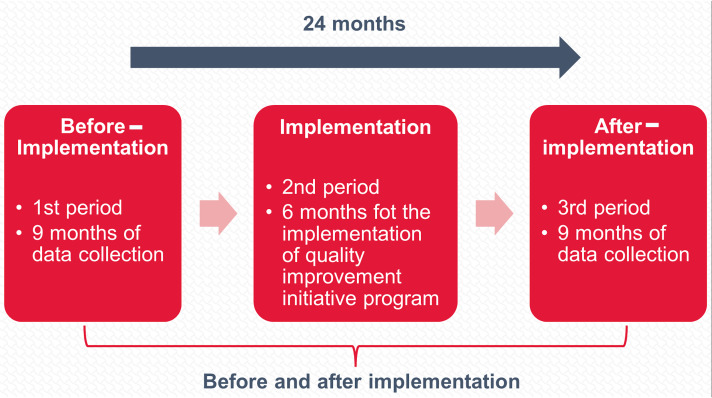


**Figure:**