# Supplementary material for: Data-driven coaching to improve statewide outcomes in CABG: before and after interventional study
Source: Int J Surg. 2024 Feb 13;110(5):2535–44. doi: 10.1097/JS9.0000000000001153 (PMC11093505; doi:10.1097/JS9.0000000000001153)
Supplement: Supplementary file 2 [file js9-110-2535-s003.docx]

**Table 1.** Standardized mean difference before and after propensity score matching.

| Propensity score matching variable | Standardized mean difference before propensity score matching | Standardized mean difference after propensity score matching |
| --- | --- | --- |
| Age | 0.038 | 0.054 |
| Gender | 0.060 | 0.109 |
| Hospital admission status | 0.390 | 0.000 |
| Diabetes control | 0.220 | 0.070 |
| STS mortality | -0.059 | 0.019 |

Standardized mean difference interpretation: 0-0.2: almost no difference, 0.2-0.5: small difference, 0.5-0.8: medium difference, 0.8-1: large difference.
