# Supplementary material for: Data-driven coaching to improve statewide outcomes in CABG: before and after interventional study
Source: Int J Surg. 2024 Feb 13;110(5):2535–44. doi: 10.1097/JS9.0000000000001153 (PMC11093505; doi:10.1097/JS9.0000000000001153)
Supplement: Supplementary file 3 [file js9-110-2535-s005.docx]

Suplementary table 2 - Univariate model

| **Explanatory Variable** | **Category** | **Reference** | **Coefficient** | **Standard Error of Coefficient** | **Odds Ratio (OR)** | **Confidence Interval 95%** | | **P Value** |
| --- | --- | --- | --- | --- | --- | --- | --- | --- |
| **Previous stroke** | No | Yes | -0,838 | 0,411 | 0,432 | 0,193 | 0,968 | **0,042** |
| **Cerebrovascular accident (CVA)** | Yes, ischemic or indeterminate | No | 2,449 | 0,410 | 11,580 | 5,184 | 25,871 | **<0,001** |
| **Rhythm change requiring permanent device implantation** | No | Pacemaker | -1,721 | 0,554 | 0,179 | 0,060 | 0,530 | **0,002** |
| **Porcelain aorta** | No | Yes | -1,741 | 0,462 | 0,175 | 0,071 | 0,434 | **<0,001** |
| **Cardiac Arrhythmia** | No | Yes | -1,587 | 0,380 | 0,205 | 0,097 | 0,431 | **<0,001** |
| **Internal thoracic artery used** | No | Yes | 1,235 | 0,418 | 3,439 | 1,517 | 7,799 | **0,003** |
| **Left internal thoracic artery** | No | Yes, pedicle | 1,021 | 0,425 | 2,777 | 1,207 | 6,389 | **0,016** |
| **Planned of extracorporeal circulation** | Unplanned | Planned | 3,422 | 0,827 | 30,645 | 6,063 | 154,884 | **<0,001** |
| **Cardiogenic shock** | Yes, during procedure | None | 4,102 | 0,875 | 60,464 | 10,889 | 335,732 | **<0,001** |
| **Cardiogenic shock** | Yes, <= 24 hours from the start of the procedure | None | 2,716 | 0,718 | 15,116 | 3,703 | 61,700 | **<0,001** |
| **CCS Class- 2 last weeks** | 4 | 1 | 0,824 | 0,331 | 2,279 | 1,190 | 4,363 | **0,013** |
| **Functional Class (NYHA)** | 3 | 1 | 0,810 | 0,311 | 2,247 | 1,222 | 4,131 | **0,009** |
| **Functional Class (NYHA)** | 4 | 1 | 1,365 | 0,429 | 3,916 | 1,688 | 9,081 | **0,001** |
| **Procedure Condition** | Emergency | Elective | 2,029 | 0,805 | 7,603 | 1,571 | 36,807 | **0,012** |
| **Pleural effusion with indication for drainage** | No | Yes | -1,741 | 0,462 | 0,175 | 0,071 | 0,434 | **<0,001** |
| **Diagnosis of Superficial Wound Infection** | Yes, within the first 30 days of post-operative period | No | -1,656 | 0,560 | 0,191 | 0,064 | 0,572 | **0,003** |
| **Dialysis** | No | Yes | -1,647 | 0,459 | 0,193 | 0,078 | 0,474 | **<0,001** |
| **Post-op hepatic dysfunction** | No | Yes | -2,635 | 1,230 | 0,072 | 0,006 | 0,800 | **0,032** |
| **Dyslipidemia** | No | Yes | 0,521 | 0,234 | 1,684 | 1,065 | 2,664 | **0,026** |
| **Peripheral arterial disease** | No | Yes | -0,846 | 0,325 | 0,429 | 0,227 | 0,812 | **0,009** |
| **Lung disease** | Severe | No | 2,508 | 0,700 | 12,286 | 3,113 | 48,484 | **<0,001** |
| **Gastro-intestinal event** | No | Yes | -1,214 | 0,448 | 0,297 | 0,123 | 0,715 | **0,007** |
| **Closure of the sternum scheduled for a second moment (Open Chest)** | No | Yes | -3,241 | 0,569 | 0,039 | 0,013 | 0,119 | **<0,001** |
| **Post-op atrial fibrillation** | No | Yes | -1,719 | 0,238 | 0,179 | 0,112 | 0,286 | **<0,001** |
| **ICU readmission** | No | Yes | -1,661 | 0,337 | 0,190 | 0,098 | 0,367 | **<0,001** |
| **Was there a need for re-intubation during the hospital stay?** | No | Yes | -4,284 | 0,294 | 0,014 | 0,008 | 0,025 | **<0,001** |
| **Deep infection/mediastinitis** | Yes, within the first 30 days of post-operative period | No | 1,356 | 0,420 | 3,881 | 1,703 | 8,844 | **0,001** |
| **Intravenous inotropes (within 48 hs)** | No | Yes | -1,964 | 0,656 | 0,140 | 0,039 | 0,508 | **0,003** |
| **Sternal instability without infectious signs** | No | Yes | -1,976 | 0,572 | 0,139 | 0,045 | 0,425 | **<0,001** |
| **Heart failure (within the last two weeks)** | No | Yes | -0,894 | 0,291 | 0,409 | 0,231 | 0,723 | **0,002** |
| **Renal insufficiency** | No | Yes | -3,003 | 0,249 | 0,050 | 0,030 | 0,081 | **<0,001** |
| **Renal insufficiency** | No | Chronic | -1,310 | 0,295 | 0,270 | 0,151 | 0,481 | **<0,001** |
| **Acute limb ischemia** | No | Yes | -3,342 | 1,007 | 0,035 | 0,005 | 0,255 | **<0,001** |
| **Need for intra-aortic balloon?** | No | Yes | -2,697 | 0,267 | 0,067 | 0,040 | 0,114 | **<0,001** |
| **Need for Post-op Dialysis** | No | Yes | -4,328 | 0,346 | 0,013 | 0,007 | 0,026 | **<0,001** |
| **Need for ReOperation due to bleeding with or without cardiac tamponade** | No | Yes | -2,152 | 0,523 | 0,116 | 0,042 | 0,324 | **<0,001** |
| **Intravenous nitrate (in the last 24 hours before surgery)** | No | Yes | -1,171 | 0,355 | 0,310 | 0,155 | 0,623 | **<0,001** |
| **Nitrates (use for more than 2 weeks before surgery)** | No | Yes | -0,547 | 0,263 | 0,579 | 0,346 | 0,969 | **0,037** |
| **Aortic occlusion** | Balloon occlusion | Total aortic clamping | 3,417 | 1,420 | 30,484 | 1,885 | 493,033 | **0,016** |
| **Aortic occlusion** | None, heart beating | Total aortic clamping | 2,031 | 0,801 | 7,621 | 1,585 | 36,632 | **0,011** |
| **Cardiac arrest** | No | Yes | -5,448 | 0,363 | 0,004 | 0,002 | 0,009 | **<0,001** |
| **Paralysis of the lower extremities** | No | Yes | -3,329 | 1,419 | 0,036 | 0,002 | 0,578 | **0,019** |
| **Paresis** | No | Yes | -1,633 | 0,778 | 0,195 | 0,043 | 0,897 | **0,036** |
| **Post-op pericardiocentesis** | No | Yes | -3,329 | 1,419 | 0,036 | 0,002 | 0,578 | **0,019** |
| **Period** | Post-implementation period | Pre-implementation period | -0,500 | 0,240 | 0,607 | 0,379 | 0,971 | **0,037** |
| **Intra-aortic balloon placement period** | Intraoperative | Preoperative | 2,642 | 0,653 | 14,048 | 3,906 | 50,522 | **<0,001** |
| **Intra-aortic balloon placement period** | post-operative | Preoperative | 2,132 | 0,600 | 8,429 | 2,601 | 27,311 | **<0,001** |
| **Platelets (un)post** | 2 | 0 | 1,779 | 0,782 | 5,923 | 1,278 | 27,452 | **0,023** |
| **Plasma (un)post** | 1 | 0 | 1,300 | 0,619 | 3,671 | 1,092 | 12,339 | **0,036** |
| **Plasma (un)post** | 2 | 0 | 1,524 | 0,638 | 4,588 | 1,313 | 16,036 | **0,017** |
| **Pneumonia** | Remote | Recent | 1,828 | 0,791 | 6,219 | 1,320 | 29,291 | **0,021** |
| **post-operative pneumonia** | No | Yes | -1,743 | 0,327 | 0,175 | 0,092 | 0,332 | **<0,001** |
| **Problems related to sternotomy** | No | Yes | -1,816 | 0,340 | 0,163 | 0,084 | 0,317 | **<0,001** |
| **Myocardial protection** | Both | Blood | 1,612 | 0,774 | 5,012 | 1,098 | 22,870 | **0,037** |
| **Clinical picture at the time of surgery** | Acute myocardial infarction with OSH | No symptoms | 2,067 | 0,652 | 7,899 | 2,200 | 28,358 | **0,002** |
| **Clinical picture at the time of surgery** | Other | No symptoms | 2,261 | 0,295 | 9,594 | 5,384 | 17,095 | **<0,001** |
| **Reasons for emergency/urgency** | IABP (intra-aortic balloon) | Acute myocardial infarction | 3,386 | 1,039 | 29,538 | 3,855 | 226,363 | **0,001** |
| **Reasons for emergency/urgency** | Other | Acute myocardial infarction | 2,133 | 0,850 | 8,440 | 1,595 | 44,647 | **0,012** |
| **ReOperation for other cardiac reasons** | No | Yes | -2,353 | 0,596 | 0,095 | 0,030 | 0,306 | **<0,001** |
| **ReOperation for other non-cardiac reasons** | No | Yes | -2,287 | 0,341 | 0,102 | 0,052 | 0,198 | **<0,001** |
| **Sepsis** | No | Yes | -2,877 | 0,272 | 0,056 | 0,033 | 0,096 | **<0,001** |
| **Stent** | No | Yes | -0,776 | 0,290 | 0,460 | 0,261 | 0,813 | **0,007** |
| **Time Between MI and Surgery** | 8 to 21 days | <= 6 hours | -3,346 | 1,459 | 0,035 | 0,002 | 0,615 | **0,022** |
| **Time Between MI and Surgery** | > 21 days | <= 6 hours | -3,513 | 1,430 | 0,030 | 0,002 | 0,491 | **0,014** |
| **Graft** | left internal thoracic artery in situ | Saphenous vein | -0,536 | 0,261 | 0,585 | 0,351 | 0,976 | **0,040** |
| **Intraoperative platelet transfusion (units)** | 1 | 0 | 1,457 | 0,706 | 4,292 | 1,075 | 17,127 | **0,039** |
| **Intraoperative platelet transfusion (units)** | 8 | 0 | 3,131 | 1,241 | 22,889 | 2,010 | 260,646 | **0,012** |
| **Intraoperative plasma transfusion (units)** | 2 | 0 | 1,593 | 0,633 | 4,921 | 1,424 | 16,999 | **0,012** |
| **Prolonged pulmonary ventilation (time > 24h)** | No | Yes | -3,488 | 0,265 | 0,031 | 0,018 | 0,051 | **<0,001** |
| **Total albumin (g/L)** |  |  | -1,027 | 0,412 | 0,358 | 0,160 | 0,803 | **0,013** |
| **Height (cm)** | No |  | -0,032 | 0,013 | 0,968 | 0,943 | 0,994 | **0,016** |
| **Creatinine clearance** | Yes, hemorrhagic |  | -0,037 | 0,011 | 0,964 | 0,943 | 0,986 | **0,001** |
| **Post-operative creatinine** | No |  | 0,381 | 0,040 | 1,463 | 1,352 | 1,583 | **<0,001** |
| **Duration of post-operative intubation** | No |  | 0,125 | 0,030 | 1,133 | 1,069 | 1,202 | **<0,001** |
| **Left ventricular ejection fraction (%)** |  |  | -0,015 | 0,006 | 0,985 | 0,972 | 0,997 | **0,018** |
| **Higher intraoperative blood glucose** |  |  | 0,004 | 0,002 | 1,004 | 1,000 | 1,007 | **0,032** |
| **Hematocrit (%)** |  |  | -0,070 | 0,021 | 0,932 | 0,894 | 0,972 | **<0,001** |
| **Lower intraoperative hematocrit** |  |  | -0,077 | 0,023 | 0,926 | 0,885 | 0,968 | **<0,001** |
| **Hematocrit at discharge** |  |  | -0,099 | 0,025 | 0,905 | 0,862 | 0,951 | **<0,001** |
| **Hemoglobin (mg/dL)** |  |  | -0,263 | 0,066 | 0,769 | 0,676 | 0,874 | **<0,001** |
| **Lower intraoperative haemoglobin** |  |  | -0,266 | 0,074 | 0,766 | 0,663 | 0,886 | **<0,001** |
| **Hemoglobin on the rise** |  |  | -0,420 | 0,110 | 0,657 | 0,530 | 0,815 | **<0,001** |
| **Age** |  |  | 0,080 | 0,014 | 1,083 | 1,053 | 1,114 | **<0,001** |
| **Weight (kg)** |  |  | -0,022 | 0,009 | 0,978 | 0,961 | 0,996 | **0,016** |
| **CKMB Peak** |  |  | 0,001 | 0,000 | 1,001 | 1,000 | 1,002 | **0,044** |
| **Blood glucose peak in 18-24 hours after the end of anesthesia** |  |  | 0,006 | 0,002 | 1,006 | 1,002 | 1,010 | **0,003** |
| **Troponin Peak I** |  |  | 0,002 | 0,001 | 1,002 | 1,001 | 1,003 | **0,001** |
| **STS mortality** |  |  | 0,690 | 0,086 | 1,994 | 1,684 | 2,361 | **<0,001** |
| **Anoxia time (min)** |  |  | 0,018 | 0,005 | 1,018 | 1,008 | 1,029 | **<0,001** |
| **CPB time (min)** |  |  | 0,021 | 0,004 | 1,021 | 1,014 | 1,028 | **<0,001** |
| **Length of ICU stay (hours)** |  |  | 0,013 | 0,004 | 1,014 | 1,006 | 1,021 | **<0,001** |
| **Total pulmonary ventilation time (in the post-operative period)** |  |  | 0,178 | 0,024 | 1,194 | 1,138 | 1,253 | **<0,001** |
| **Lowest creatinine (mg/dL)** |  |  | 0,250 | 0,056 | 1,284 | 1,150 | 1,432 | **<0,001** |
| **Transient ischemic attack** | No | Yes | 13,259 | 565,578 | >1000 | 0,000 | Inf | 0,981 |
| **Cerebrovascular accident (CVA)** | Yes, hemorrhagic | No | -10,136 | 535,411 | 0,000 | 0,000 | Inf | 0,985 |
| **Sleep apnea** | No | Yes | -1,128 | 1,061 | 0,324 | 0,040 | 2,588 | 0,288 |
| **Radial artery used** | No | Yes | 1,065 | 1,012 | 2,902 | 0,399 | 21,102 | 0,293 |
| **Transient ischemic attack** | No | Yes | 11,252 | 441,372 | >1000 | 0,000 | Inf | 0,980 |
| **Right Internal Thoracic Artery** | Yes, skeletonized | Yes, pedicle | -0,121 | 0,826 | 0,886 | 0,176 | 4,469 | 0,883 |
| **Right Internal Thoracic Artery** | No | Yes, pedicle | 0,458 | 0,597 | 1,581 | 0,491 | 5,093 | 0,443 |
| **Left Internal Thoracic Artery** | Yes, skeletonized | Yes, pedicle | 0,283 | 0,248 | 1,327 | 0,815 | 2,158 | 0,255 |
| **Total atrioventricular blocks** | <= 30 days preop | > 30 days preop | 13,650 | 441,372 | >1000 | 0,000 | Inf | 0,975 |
| **Total atrioventricular blocks** | No | > 30 days preop | 11,228 | 441,372 | >1000 | 0,000 | Inf | 0,980 |
| **Cancer in the last 5 years** | No | Yes | -1,039 | 0,617 | 0,354 | 0,106 | 1,186 | 0,092 |
| **Use of cardio pulmonary bypass** | Yes | No | -0,522 | 0,335 | 0,593 | 0,308 | 1,143 | 0,119 |
| **Prior Coronary Artery Bypass Graft Surgery** | No | Yes | 12,252 | 650,874 | >1000 | 0,000 | Inf | 0,985 |
| **CCS Class- 2 last weeks** | 2 | 1 | -0,141 | 0,319 | 0,869 | 0,465 | 1,623 | 0,659 |
| **CCS Class- 2 last weeks** | 3 | 1 | 0,355 | 0,314 | 1,426 | 0,771 | 2,638 | 0,258 |
| **Functional Class (NYHA)** | 2 | 1 | 0,334 | 0,316 | 1,396 | 0,751 | 2,594 | 0,291 |
| **Procedure Condition** | Urgency | Elective | 0,216 | 0,244 | 1,241 | 0,769 | 2,003 | 0,376 |
| **Colour** | Black | White | -0,327 | 0,729 | 0,721 | 0,173 | 3,011 | 0,654 |
| **Colour** | Brown | White | -0,405 | 0,347 | 0,667 | 0,338 | 1,317 | 0,244 |
| **Colour** | Yellow | White | 0,481 | 0,740 | 1,618 | 0,379 | 6,904 | 0,516 |
| **Colour** | Indigena | White | -11,282 | 624,194 | 0,000 | 0,000 | Inf | 0,986 |
| **Colour** | Unknown/Not informed | White | 1,205 | 0,627 | 3,336 | 0,977 | 11,397 | 0,055 |
| **Depression** | No | Yes | -0,021 | 0,730 | 0,979 | 0,234 | 4,094 | 0,977 |
| **Diabetes mellitus** | No | Yes | -0,188 | 0,235 | 0,828 | 0,523 | 1,312 | 0,422 |
| **Diagnosis of Superficial Wound Infection** | Yes, after 30 days of post-operative period, during hospitalization | No | -15,602 | 1495,296 | 0,000 | 0,000 | Inf | 0,992 |
| **Post-operative multi-organ dysfunction** | No | Yes | -20,152 | 565,578 | 0,000 | 0,000 | Inf | 0,972 |
| **Percutaneous MAD device** | No | Yes | 11,252 | 441,372 | >1000 | 0,000 | Inf | 0,980 |
| **Carebrovascular disease** | No | Yes | -0,371 | 0,334 | 0,690 | 0,359 | 1,328 | 0,267 |
| **Thoracic aortic disease** | No | Yes | -1,380 | 1,075 | 0,252 | 0,031 | 2,071 | 0,199 |
| **Sinus node disease** | No | <= 30 days preop | 10,250 | 535,411 | >1000 | 0,000 | Inf | 0,985 |
| **Liver disease** | No | Yes | 0,327 | 1,020 | 1,386 | 0,188 | 10,232 | 0,749 |
| **Lung disease** | Lightweight | No | -13,210 | 619,560 | 0,000 | 0,000 | Inf | 0,983 |
| **Lung disease** | Moderate | No | 1,276 | 1,067 | 3,583 | 0,442 | 29,033 | 0,232 |
| **Rheumatic heart disease** | No | Yes | 12,254 | 485,133 | >1000 | 0,000 | Inf | 0,980 |
| **ECMO** | Veno-arterial | Veno-venous | -31,132 | 1328,590 | 0,000 | 0,000 | Inf | 0,981 |
| **ECMO** | No | Veno-venous | -18,907 | 1029,121 | 0,000 | 0,000 | Inf | 0,985 |
| **Endocarditis** | No | Yes | -16,895 | 535,411 | 0,000 | 0,000 | Inf | 0,975 |
| **Cardioplegia Delivery** | Anterograda | No | 0,916 | 1,015 | 2,499 | 0,342 | 18,273 | 0,367 |
| **Cardioplegia Delivery** | Retrograde | No | 1,974 | 1,454 | 7,200 | 0,417 | 124,433 | 0,175 |
| **Cardioplegia Delivery** | Both | No | -10,289 | 624,195 | 0,000 | 0,000 | Inf | 0,987 |
| **Venous Grafts Used** | No | Yes | -0,200 | 0,380 | 0,819 | 0,389 | 1,723 | 0,599 |
| **Schooling** | Elementary (primary education) | Illiterate | 0,106 | 0,748 | 1,112 | 0,257 | 4,812 | 0,888 |
| **Schooling** | High School (Secondary Education) | Illiterate | -1,244 | 0,928 | 0,288 | 0,047 | 1,775 | 0,180 |
| **Schooling** | Superior | Illiterate | -0,940 | 0,928 | 0,391 | 0,063 | 2,410 | 0,311 |
| **Schooling** | Unknown/Not available | Illiterate | -0,239 | 0,742 | 0,787 | 0,184 | 3,368 | 0,747 |
| **Carotid stenosis** | Right | No | 0,189 | 0,930 | 1,208 | 0,195 | 7,485 | 0,839 |
| **Carotid stenosis** | Left | No | 0,574 | 0,836 | 1,776 | 0,345 | 9,136 | 0,492 |
| **Carotid stenosis** | Of both | No | 0,940 | 0,841 | 2,559 | 0,492 | 13,306 | 0,264 |
| **Carotid stenosis** | Undocumented | No | 0,005 | 0,601 | 1,005 | 0,310 | 3,261 | 0,994 |
| **Extubation in the operating room** | No | Yes | 0,460 | 0,596 | 1,584 | 0,493 | 5,096 | 0,440 |
| **Atrial fibrillation** | <= 30 days preop | >30 days preop | 1,124 | 1,195 | 3,077 | 0,296 | 31,984 | 0,347 |
| **Atrial fibrillation** | No | >30 days preop | -1,071 | 1,056 | 0,343 | 0,043 | 2,715 | 0,311 |
| **Atrial flutter** | <= 30 days preop | >30 days preop | 0,000 | 1188,327 | 1,000 | 0,000 | Inf | 1,000 |
| **Atrial flutter** | No | >30 days preop | 12,253 | 840,274 | >1000 | 0,000 | Inf | 0,988 |
| **Hypertension** | No | Yes | 0,423 | 0,322 | 1,527 | 0,812 | 2,872 | 0,189 |
| **Family history of coronary heart disease** | No | Yes | 0,460 | 0,379 | 1,584 | 0,754 | 3,327 | 0,224 |
| **ICP - Did you use a stent?** | No | Yes | -0,434 | 0,312 | 0,648 | 0,352 | 1,195 | 0,165 |
| **ICP made during admission** | Yes, in another hospital | Yes, in this hospital | -14,081 | 619,560 | 0,000 | 0,000 | Inf | 0,982 |
| **ICP made during admission** | No | Yes, in this hospital | -0,855 | 0,534 | 0,425 | 0,149 | 1,212 | 0,110 |
| **Current immunosuppression** | No | Yes | 13,257 | 619,560 | >1000 | 0,000 | Inf | 0,983 |
| **Previous myocardial infarction** | No | Yes | 0,004 | 0,234 | 1,004 | 0,635 | 1,587 | 0,988 |
| **Surgical site infection within 30 days of procedure** | No | Yes | -0,348 | 0,303 | 0,706 | 0,390 | 1,278 | 0,250 |
| **Surgical site infection: Thoracotomy** | Yes, within the first 30 days of post-operative period | No | 0,330 | 0,406 | 1,391 | 0,628 | 3,083 | 0,416 |
| **Surgical site infection: Thoracotomy** | Yes, after 30 days of post-operative period, during hospitalization | No | -12,226 | 650,874 | 0,000 | 0,000 | Inf | 0,985 |
| **Infection at the graft dissection site** | Yes, within the first 30 days of post-operative period | No | -0,087 | 0,470 | 0,917 | 0,365 | 2,306 | 0,854 |
| **Infection at the graft dissection site** | Yes, after 30 days of post-operative period, during hospitalization | No | -12,258 | 650,874 | 0,000 | 0,000 | Inf | 0,985 |
| **Deep infection/mediastinitis** | Yes, after 30 days of post-operative period, during hospitalization | No | 2,013 | 1,125 | 7,485 | 0,826 | 67,868 | 0,074 |
| **Renal insufficiency** | Acute | Chronic | 0,118 | 0,795 | 1,125 | 0,237 | 5,344 | 0,882 |
| **Interval between PCI and surgery** | > 6 hours | <= 6 hours | 13,638 | 1696,734 | >1000 | 0,000 | Inf | 0,994 |
| **Prior cardiac intervention** | No | Yes | 12,254 | 514,561 | >1000 | 0,000 | Inf | 0,981 |
| **Prior percutaneous coronary intervention (PCI)** | No | Yes | -0,449 | 0,295 | 0,638 | 0,358 | 1,138 | 0,128 |
| **Need for ReOperation for Myocardial Ischemia** | No | Yes | 11,251 | 624,194 | >1000 | 0,000 | Inf | 0,986 |
| **Number of vessels affected** | One | None | -17,829 | 535,412 | 0,000 | 0,000 | Inf | 0,973 |
| **Number of vessels affected** | Two | None | -17,035 | 535,411 | 0,000 | 0,000 | Inf | 0,975 |
| **Number of vessels affected** | Three | None | -16,833 | 535,411 | 0,000 | 0,000 | Inf | 0,975 |
| **Aortic occlusion** | None, fibrillating heart | Total aortic clamping | -10,149 | 535,411 | 0,000 | 0,000 | Inf | 0,985 |
| **Other Antiplatelet Agent** | No | Yes | 13,259 | 565,578 | >1000 | 0,000 | Inf | 0,981 |
| **Other non-cardiac procedure performed** | No | Yes | 12,254 | 514,561 | >1000 | 0,000 | Inf | 0,981 |
| **Percutaneous MAD Installation Period** | Intraoperative | Preoperative | 0,000 | 79462,005 | 1,000 | 0,000 | Inf | 1,000 |
| **Pneumonia** | No | Recent | -13,234 | 641,305 | 0,000 | 0,000 | Inf | 0,984 |
| **Pneumothorax with indication for intervention** | No | Yes | -1,031 | 0,751 | 0,357 | 0,082 | 1,555 | 0,170 |
| **Post Liver Transplantation** | No | Yes | 11,251 | 624,194 | >1000 | 0,000 | Inf | 0,986 |
| **Myocardial protection** | Crystalloid | Blood | 0,479 | 0,297 | 1,614 | 0,901 | 2,891 | 0,108 |
| **Myocardial protection** | Other | Blood | 0,306 | 1,032 | 1,357 | 0,180 | 10,256 | 0,767 |
| **Clinical status at the time of surgery** | Stable angina | No symptoms | -0,724 | 1,017 | 0,485 | 0,066 | 3,558 | 0,477 |
| **Clinical status at the time of surgery** | Unstable angina | No symptoms | -0,004 | 1,024 | 0,996 | 0,134 | 7,406 | 0,997 |
| **Clinical status at the time of surgery** | Non-OSH Acute myocardial infarction | No symptoms | 0,494 | 0,737 | 1,638 | 0,387 | 6,942 | 0,503 |
| **Clinical status at the time of surgery** | Ischemic equivalent | No symptoms | -11,959 | 650,874 | 0,000 | 0,000 | Inf | 0,985 |
| **Mediastinal radiotherapy** | No | Yes | 10,250 | 535,411 | >1000 | 0,000 | Inf | 0,985 |
| **Reasons for emergency/urgency** | Intercardiac mass or thrombus | Acute myocardial infarction (acute infarction) | -13,180 | 2399,545 | 0,000 | 0,000 | Inf | 0,996 |
| **Reasons for emergency/urgency** | Ongoing ischemia | Acute myocardial infarction (acute infarction) | -0,945 | 1,045 | 0,389 | 0,050 | 3,016 | 0,366 |
| **Reasons for emergency/urgency** | Incomplete PCI without clinical deterioration | Acute myocardial infarction (acute infarction) | -13,180 | 2399,545 | 0,000 | 0,000 | Inf | 0,996 |
| **Reasons for emergency/urgency** | PCI or attempted PCI with clinical deterioration | Acute myocardial infarction (acute infarction) | 1,999 | 1,153 | 7,385 | 0,771 | 70,766 | 0,083 |
| **Reasons for emergency/urgency** | Pulmonary edema | Acute myocardial infarction (acute infarction) | -13,180 | 2399,545 | 0,000 | 0,000 | Inf | 0,996 |
| **Reasons for emergency/urgency** | Angina at rest | Acute myocardial infarction (acute infarction) | 0,053 | 0,535 | 1,055 | 0,369 | 3,013 | 0,920 |
| **Reasons for emergency/urgency** | Shock with circulatory support | Acute myocardial infarction (acute infarction) | -13,180 | 2399,545 | 0,000 | 0,000 | Inf | 0,996 |
| **Reasons for emergency/urgency** | Anatomy | Acute myocardial infarction (acute infarction) | 0,468 | 0,779 | 1,597 | 0,347 | 7,348 | 0,548 |
| **Reasons for emergency/urgency** | Syncope | Acute myocardial infarction (acute infarction) | 1,776 | 1,131 | 5,908 | 0,644 | 54,235 | 0,116 |
| **Reasons for emergency/urgency** | Worsening of cardiopulmonary status | Acute myocardial infarction (acute infarction) | 1,034 | 0,792 | 2,813 | 0,596 | 13,283 | 0,192 |
| **Reasons for emergency/urgency** | Aortic aneurysm | Acute myocardial infarction (acute infarction) | -13,180 | 2399,545 | 0,000 | 0,000 | Inf | 0,996 |
| **Reasons for emergency/urgency** | Congestive heart failure | Acute myocardial infarction (acute infarction) | 1,188 | 1,091 | 3,282 | 0,387 | 27,859 | 0,276 |
| **Reasons for emergency/urgency** | Complications with diagnostic/interventional procedure | Acute myocardial infarction (acute infarction) | -13,180 | 1696,734 | 0,000 | 0,000 | Inf | 0,994 |
| **Readmission within 30 days** | No | Yes | 14,287 | 453,805 | >1000 | 0,000 | Inf | 0,975 |
| **Aortic reintervention** | No | Yes | -16,895 | 535,411 | 0,000 | 0,000 | Inf | 0,975 |
| **Prior carotid revascularization (surgery or stenting)** | No | Yes | -0,519 | 0,606 | 0,595 | 0,181 | 1,953 | 0,392 |
| **Sex** | Female | Male | 0,293 | 0,255 | 1,341 | 0,814 | 2,209 | 0,250 |
| **Syncope** | No | Yes | 0,544 | 1,017 | 1,722 | 0,234 | 12,648 | 0,593 |
| **Cancer status** | Active treatment | Remission | -1,465 | 0,769 | 0,231 | 0,051 | 1,043 | 0,057 |
| **Smoking** | Current everyday | Never | -0,277 | 0,449 | 0,758 | 0,315 | 1,827 | 0,537 |
| **Smoking** | Current but not every day | Never | -12,158 | 550,089 | 0,000 | 0,000 | Inf | 0,982 |
| **Smoking** | Former smoker | Never | 0,316 | 0,251 | 1,371 | 0,838 | 2,243 | 0,209 |
| **Time Between MI and Surgery** | > 6 < 24 hours | <= 6 hours | -15,566 | 840,275 | 0,000 | 0,000 | Inf | 0,985 |
| **Time Between MI and Surgery** | 1 to 7 days | <= 6 hours | -2,526 | 1,461 | 0,080 | 0,005 | 1,402 | 0,084 |
| **Type of admission** | Emergency / Urgency | Elective | 0,362 | 0,268 | 1,436 | 0,849 | 2,430 | 0,178 |
| **Type of admission** | Transfer from another Hospital | Elective | -0,010 | 0,317 | 0,991 | 0,533 | 1,842 | 0,976 |
| **Type of atrial fibrillation** | Persistent (duration > 7 days) | Paroxysmal (self-limiting, duration < 7 days) | -0,357 | 1,233 | 0,700 | 0,062 | 7,853 | 0,772 |
| **Type of atrial fibrillation** | Long-lasting persistent (duration >= 1 year) | Paroxysmal (self-limiting, duration < 7 days) | -17,313 | 4612,202 | 0,000 | 0,000 | Inf | 0,997 |
| **Type of atrial fibrillation** | Permanent (failure to reverse the arrhythmia) | Paroxysmal (self-limiting, duration < 7 days) | -17,313 | 4612,202 | 0,000 | 0,000 | Inf | 0,997 |
| **Diabetes Treatment** | Oral Hypoglycemic | No control | 15,349 | 2662,854 | >1000 | 0,000 | Inf | 0,995 |
| **Diabetes Treatment** | Insulin | No control | 15,511 | 2662,854 | >1000 | 0,000 | Inf | 0,995 |
| **Deep vein thrombosis** | No | Yes | 11,251 | 624,194 | >1000 | 0,000 | Inf | 0,986 |
| **Ventricular tachycardia/ventricular fibrillation** | <= 30 days preop | >30 days preop | -16,646 | 624,195 | 0,000 | 0,000 | Inf | 0,979 |
| **Ventricular tachycardia/ventricular fibrillation** | No | >30 days preop | -17,919 | 624,194 | 0,000 | 0,000 | Inf | 0,977 |
| **Single-vessel or multi-vessel** | Multivessel | Single-vessel | -16,093 | 1684,138 | 0,000 | 0,000 | Inf | 0,992 |
| **Alcohol use** | 2-7 servings per week | <= 1 serving per week | -0,862 | 0,817 | 0,422 | 0,085 | 2,094 | 0,291 |
| **Alcohol use** | >= 8 servings per week | <= 1 serving per week | -0,094 | 0,827 | 0,911 | 0,180 | 4,606 | 0,910 |
| **Alcohol use** | None | <= 1 serving per week | -0,691 | 0,411 | 0,501 | 0,224 | 1,120 | 0,092 |
| **Illicit drug use** | Recent | No | -12,255 | 650,874 | 0,000 | 0,000 | Inf | 0,985 |
| **Illicit drug use** | Remote | No | -12,255 | 594,164 | 0,000 | 0,000 | Inf | 0,984 |
| **Duration of surgery (hours)** |  |  | 0,104 | 0,085 | 1,109 | 0,940 | 1,310 | 0,220 |
| **Post-procedure left ventricular ejection fraction (%)** |  |  | 0,000 | 14647,441 | 1,000 | 0,000 | Inf | 1,000 |
| **Blood glucose (mg/dL)** |  |  | 0,000 | 0,002 | 1,000 | 0,996 | 1,005 | 0,841 |
| **Glycosylated haemoglobin** |  |  | 0,067 | 0,096 | 1,069 | 0,886 | 1,290 | 0,487 |
| **Additional hours of post-operative ventilation** |  |  | 0,002 | 0,002 | 1,002 | 0,998 | 1,006 | 0,345 |
| **Body mass index** |  |  | -0,033 | 0,029 | 0,968 | 0,914 | 1,025 | 0,261 |
| **Lower temperature** |  |  | 0,020 | 0,070 | 1,020 | 0,890 | 1,170 | 0,776 |
| **Length of post-operative stay (days)** |  |  | -0,018 | 0,046 | 0,982 | 0,898 | 1,074 | 0,688 |
| **Total length of hospital stay (days)** |  |  | -0,015 | 0,027 | 0,985 | 0,934 | 1,039 | 0,574 |
